# Detecting functional rare variants by collapsing and incorporating functional annotation in Genetic Analysis Workshop 17 mini-exome data

**DOI:** 10.1186/1753-6561-5-S9-S27

**Published:** 2011-11-29

**Authors:** Xiting Yan, Lun Li, Joon Sang Lee, Wei Zheng, John Ferguson, Hongyu Zhao

**Affiliations:** 1 Department of Epidemiology and Public Health, Yale University, New Haven, CT 06520, USA; 2 Hubei Bioinformatics and Molecular Imaging Key Laboratory, Huazhong University of Science and Technology, Wuhan, Hubei, China; 3 Keck Laboratory, Yale University, New Haven, CT 06511, USA

## Abstract

Association studies using tag SNPs have been successful in detecting disease-associated common variants. However, common variants, with rare exceptions, explain only at most 5–10% of the heritability resulting from genetic factors, which leads to the common disease/rare variants assumption. Indeed, recent studies using sequencing technologies have demonstrated that common diseases can be due to rare variants that could not be systematically studied earlier. Unfortunately, methods for common variants are not optimal if applied to rare variants. To identify rare variants that affect disease risk, several investigators have designed new approaches based on the idea of collapsing different rare variants inside the same genomic block (e.g., the same gene or pathway) to enrich the signal. Here, we consider three different collapsing methods in the multimarker regression model and compared their performance on the Genetic Analysis Workshop 17 data using the consistency of results across different simulations and the cross-validation prediction error rate. The comparison shows that the proportion collapsing method seems to outperform the other two methods and can find both truly associated rare and common variants. Moreover, we explore one way of incorporating the functional annotations for the variants in the data that collapses nonsynonymous and synonymous variants separately to allow for different penalties on them. The incorporation of functional annotations led to higher sensitivity and specificity levels when the detection results were compared with the answer sheet. The initial analysis was performed without knowledge of the simulating model.

## Background

Genome-wide association studies (GWAS) have successfully identified thousands of common variants associated with the risk of common diseases [1,2]. To date, GWAS have been mostly conducted under the common disease/common variants (CDCV) hypothesis, which asserts that common diseases are mostly caused by common variants with small to modest effects [3-6]. Typically, only variants with a minor allele frequency (MAF) greater than 1–5% are considered in these studies. However, despite the identification of thousands of common variants that affect common disease risk, with rare exceptions these common variants can explain at most 5–10% of the heritable component of disease [7]. Theoretical studies based on evolutionary theories suggest that less common variations are more likely to be functional than common variations [8,9]. Recent studies using sequencing technology have also detected many rare variants that are associated with disease [7], providing empirical evidence for the common disease/rare variant (CDRV) hypothesis. All these studies suggest that the complex disease etiology can be a mixture of common variants and rare variants.

Typical GWAS detect disease-associated variants using indirect linkage disequilibrium (LD) mapping, which captures the information of correlated single-nucleotide polymorphisms (SNPs) using a set of tag SNPs to reduce the number of testing. However, this strategy is not efficient when applied to rare variants because the correlation between the rare variants and the tag SNPs is often weak as a result of the low MAF of the rare variants. Alternative LD measures for fine mapping have been developed and offer some advantages over the traditional LD mapping [10]. In addition, direct mapping through exhaustive genotyping or sequencing is more appropriate for identifying functional rare variants.

To analyze the sequencing data, many investigators have developed association tests to detect disease-associated rare variants. These tests fall into three main types: (1) multiple univariate single-marker tests, (2) multiple-marker tests, and (3) collapsing methods. The univariate single-marker tests assess the significance of association for every rare variant independently. The multiple-marker tests instead test for the association of a set of variants simultaneously [11]. Both single-marker and multiple-marker tests have reduced power because of the multiple testing correction. In addition, the power of single-marker tests for low-frequency variants is sensitive to the effect size [12]. The collapsing methods combine information across multiple variants in the same genomic block (e.g., the same gene or pathway) so that the association signals can be enriched and the test’s degrees of freedom can be reduced [11-14].

Here, we consider three different collapsing methods for rare variants in the same gene. Regression with a LASSO (least absolute shrinkage and selection operator) penalty is then used to choose the significant collapsed rare variants or common variants. The three collapsing methods are compared based on the consistency across replicates, the cross-validation error rate of the fitted model, and the list of true causal variants. The most significant common variants and collapsed rare variants are shown. We also explore the incorporation of the functional annotation information of all the variants in the regression model. By comparing the results with the list of true causal variants, we find that incorporation of the functional annotation leads to higher sensitivity and specificity levels.

## Methods

### Collapsing rare variants

All the variants are divided into two groups. Variants with MAF > 5% fall into the common variants group, and all the other variants form the rare variants group. Note that this definition of rare variants is specific to this paper. We also considered a more common definition of rare variants with MAF ≤ 1% and came to the same conclusions (results not shown). The rare variants in the same gene are collapsed using the proportion coding (PROP), the data-adaptive sum (DAS), and the weighted-sum (WS) methods. Details and assumptions of these collapsing methods can be found in Dering et al. [15].

### Multiple regression model

We used a multiple regression model to assess the association of variants with the phenotype after the collapsing. Suppose that in individual *i* the collapsed genetic score is  for gene *l* and  for common variant *v_j_*. Note that for the weighted sum collapsing method,  for all the common variants because common and rare variants are collapsed into one single term. Let *G* = (*g*_1_, *g*_2_, …, *g_L_*) denote the set of all the genes and *CV* = (*v*_1_, *v*_2_, …, *v_M_*) denote the set of all common variants. If *Y_i_* is the disease status or the trait value of individual *i*, then the multiple regression model is:(1)

where *E_i_* is the vector of the environmental variables for individual *i*, *β_E_* is the vector of coefficients for these variables, *g*(·) is the link function, and *μ_i_* is the mean of *Y_i_*. For binary disease status we use the logit link function, and for the other three quantitative trait models we use the identity link function. For parameter estimation, we use a least absolute shrinkage and selection operator (LASSO) [16], which penalizes the likelihood function by adding the sum of the absolute value of the coefficients (L1 penalty function). Many of the coefficients will be shrunk to 0 as a result of the property of the L1 penalty function.

### Comparing collapsing methods

The Genetic Analysis Workshop 17 (GAW17) data contain 200 simulations, and we treat them as replicates. We use each of the replicates to fit the regression model using a LASSO. Genes with nonzero estimated coefficients are taken to be identified. The consistency of the identified genes across replicates by each method is measured in the following way. Suppose that in the fitted model for replicate *i*, *s_ij_* = 1 if gene *j* has a nonzero coefficient and 0 otherwise. Then the consistency of the method across different replicates is measured by:(2)

where *F* contains all the genes identified by the model fitted in at least one replicate data set and |*F*| is the size of *F*. The three collapsing methods are compared based on this consistency score. The ability of the consistency score to evaluate the performance of the collapsing methods is debatable because a method can be consistently bad but have a good consistency score. Therefore we further compare the three collapsing methods using the cross-validation error rate of the fitted model. We fit one model for each of the 200 replicates and use the fitted model to predict the trait values in the other 199 replicates. The prediction is then compared with the true values to calculate the error rate. For the disease trait, an area under curve (AUC) score is calculated for each of the 199 validation replicates and the average AUC score is returned, whereas for quantitative traits the mean-square error is used as the measure of prediction error.

### Incorporating functional annotation

Mutations in the coding region that change the function of the encoded proteins or that fall into highly conserved regions tend to affect the biological function significantly. For each SNP in the data, the functional annotation describes whether the SNP is nonsynonymous or synonymous. We try to incorporate this functional annotation information by collapsing the nonsynonymous and synonymous SNPs separately and shrinking their parameters to different extents to allow different probabilities of association. The modified model can be formulated as:(3)

where  and  are the parameters for the collapsed nonsynonymous and synonymous rare variants in gene *g_l_*, respectively. To shrink the parameters for the synonymous and nonsynonymous variants to different extents, the penalized log-likelihood function is set as:(4)

where *l*(*β*) is the log-likelihood function, *a_j_* = *ns* indicates that the corresponding variant is nonsynonymous, and *a_j_* = *s* indicates that the variant is synonymous. The two penalty parameters *λ_ns_* and *λ_s_* are chosen based on the cross-validation error rate within each replicate data set.

## Results

### Comparison of collapsing methods

Two hundred simulations in the GAW17 data set [17] provide values of four traits, including disease, Q1, Q2, and Q4. We treat these 200 simulations as replicates. For each replicate, we fit a multiple regression model using a LASSO. We measure the consistency of the significant features across the 200 replicates by using the score defined in Eq. (2); the comparison of the consistency between the three collapsing methods is shown in Table 1. For the disease model, the proportion collapsing method achieves the most consistent results across replicates. For the other three traits, the proportion collapsing method also has the best consistency.

**Table 1:** Consistency scores of the selected features from the 200 replicates using the three different collapsing methods

Method	Q1	Q2	Q4	Disease
Proportion collapsing	475.7897	316.2296	291.7924	344.5995
Data-adaptive sum	645.315	649.8706	420.0192	369.6984
Weighted sum	803.3252	366.4013	323.6185	468.9865

Alternatively, we fit one model for each replicate and use the fitted model to predict the trait values of the other 199 replicates. The improvement in the prediction accuracy resulting from the genetic features is thus obtained and is shown in Table 2. The comparisons show that the proportion collapsing method again has the best prediction accuracy for all traits except Q4. In fact, the prediction accuracy for Q4 decreases if the genetic features are included; this is due to the structure of the simulation model. Although Q4 has a heritability of 0.7, none of the heritability is due to genes in the data set. Based on the comparisons, we conclude that the proportion collapsing method achieves the most consistent results and the lowest prediction errors.

**Table 2:** Improvement in the prediction accuracy of the fitted regression model in the testing replicates using the three collapsing methods

Method	Q1 (mean-square error)	Q2 (mean-square error)	Q4 (mean-square error)	Disease (average AUC score)
Proportion collapsing	0.1226466	0.0116719	−0.0068168	0.0023503
Data-adaptive sum	0.1193559	0.0027142	−0.0080903	0.0015294
Weighted sum	0.0779981	0.0080481	−0.0060441	0.0013407

**Figure 1:**
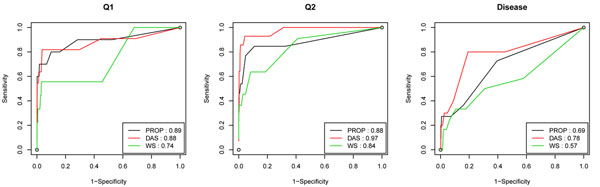
**ROC curves and AUC scores of different collapsing methods on Q1, Q2, and disease models.** PROP, proportion collapsing method; DAS, data-adaptive sum method; and WS, weighted-sum method. AUC scores are shown next to the names of the corresponding methods.

In addition, we rank genes and variants based on the number of replicates that provide nonzero parameter estimates for them. For a given threshold, the set of significant genes or variants can be defined. By comparing this list of genes with the true causal variants and genes for disease, Q1, and Q2, we calculate the sensitivity and specificity to draw the receiver operating characteristic (ROC) curves, which are shown in Figure 1. Note that sensitivity and specificity reflect the type I and II error of the method, respectively. The area under the ROC curves, defined as the AUC score, is also calculated for each method. The higher the AUC score is, the better performance the method has. Comparison of the ROC curves and AUC scores for different methods shows that for Q2 and disease, the data-adaptive collapsing method performs better than the other two methods. For Q1, however, the proportion collapsing method has the best performance.

### Identifying associated variants by incorporating functional annotations

**Figure 2:**
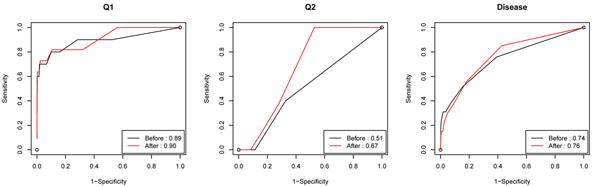
ROC curves and AUC score of results before and after incorporating the functional annotation

Intuitively, mutations in a gene that change the function of the corresponding encoded protein tend to be more deleterious. Therefore we collapse the nonsynonymous and synonymous rare variants into two different terms and allow different shrinkage for them in the LASSO model. We use the proportion collapsing method to do the collapsing because of its consistently good performance for most of the criteria and trait models. Results before and after incorporating the functional annotation information are compared with the list of truly associated SNPs to generate the ROC curves and their AUC scores, which are shown in Figure 2. The AUC score increases the most for Q2, from 0.51 to 0.67. For disease and Q1, the improvements are 0.01 and 0.02, respectively. The improvements in the AUC scores suggest that incorporating functional annotation improves the detection accuracy of the associated variants. This is consistent with the fact that in the simulation model all functional variants are nonsynonymous.

In Table 3, we show the 10 most significant features associated with the four traits. According to the table, there seem to be no environmental effects for Q2, whereas Q4 seems to be affected mostly by the Age, Sex, and Smoke covariates. The population information is included as one of the covariates. But as shown in Table 3, none of the traits rank the population variable as the top 10 most significant feature. All traits except Q4 seem to be significantly associated with variants inside some genes. Compared with the list of true causal variants and genes in the answer sheet, the LASSO regression model detects both true causal common and rare variants for Q1 and Q2 but not for disease. This is because the disease liability is decided by the three quantitative traits and a set of variants. However, in the generalized linear model, none of the quantitative traits are included as predictors, reducing the performance of the model significantly, especially when these traits are actually also decided by the variants included in the model.

**Table 3:** The 10 most significant features selected for the disease model and the three quantitative traits when rare variants are collapsed using the proportion collapsing method

Feature	Q1	Q2	Q4	Disease
1	Age (200)	C6S5380 (108)	Sex (200)	Age (200)
2	Smoke (200)	C6S5441 (51)	Age (200)	Smoke (180)
3	*FLT1* (n) (200)	*PDGFD* (n) (50)	Smoke (200)	C13S523 (39)
4	C13S523 (200)	*LPL* (n) (38)	*C1ORF122* (s) (8)	*FLT1* (n) (25)
5	*KDR* (n) (175)	*VLDLR* (n) (32)	C3S4667 (8)	*PTK7* (s) (16)
6	C4S1878 (76)	*BCHE* (n) (31)	*FLJ16793* (s) (8)	*ADCY5* (s) (12)
7	*ARNT* (n) (72)	*SIRT1* (n) (28)	*RY1* (s) (8)	*HOXD11* (s) (12)
8	*MAP2K7* (s) (62)	*TXNL1* (n) (24)	*ACOX3* (s) (7)	*TFDP1* (s) (12)
9	*NT5C2* (s) (50)	*RARB* (n) (24)	*OR13A1* (s) (7)	*OR8D4* (s) (11)
10	*FOXO3* (s) (35)	C9S3419 (22)	C14S697 (7)	*CCNT1* (s) (11)

Numbers following each feature name are the numbers of replicates that have nonzero coefficients in the fitted model. Common variants have their original name. Gene names followed by (s) stand for the collapsed synonymous rare variants in the gene, and those followed by (n) stand for the collapsed nonsynonymous rare variants in the gene.

## Discussion and conclusions

We compared three different collapsing methods using the GAW17 data and explored one way to incorporate the functional annotation information. The analysis shows that for the GAW17 data, the proportion collapsing method tends to have the best performance in terms of consistency across different simulations and cross-validation error rate. Furthermore, incorporation of the functional information leads to higher specificity and sensitivity levels. Finally, by comparing the identified genes with the true causal genes, we show that the LASSO method in combination with the rare-variants collapsing method is able to detect most of the true causal variants and genes for the three quantitative traits.

However, several issues need to be addressed with regard to the analysis. First, note that, based on both the consistency score and the cross-validation error rate, the performance of the proportion collapsing method drops when applied to Q2 and disease trait compared to Q1. In fact, Q1 is affected by the covariates Age and Smoke, which can be consistently detected easily and which cause the consistency score to be the best. For disease and Q2, this effect of the covariates is much weaker and thus leads to worse consistency. These results suggest that the consistency score may not be optimal to evaluate the performance of the collapsing methods.

Second, the improvement in the AUC score achieved by incorporating the functional annotation was not impressive for disease and Q1, given that all the functional variants in the simulation model are nonsynonymous. This again can be related to the higher residual heritability of Q1 resulting from variants not included in the data set. It also suggests that our current way of incorporating the functional annotation is not optimal.

Third, many important questions are not answered in this analysis. They include how to detect the interactions between genes and environmental variables, alternative ways to incorporate the functional annotation such as Bayesian methods with different prior probabilities for the synonymous and nonsynonymous variants, adding the quantitative traits in the disease models as predictors, and applying the generalized additive model.

## Competing interests

The authors declare that they have no competing interests.

## Authors’ contributions

XY conceived of the study, performed the data analysis and wrote the manuscript. LL, JSL and WZ participated in the data preprocessing and analysis. JF helped to draft the manuscript. All authors participated in design of the study. HZ conceived of the study, coordinated the analysis and wrote the manuscript. All authors read and approved the final manuscript.
